# WEE1 inhibition enhances sensitivity to hypoxia/reoxygenation in HeLa cells

**DOI:** 10.1093/jrr/rrz045

**Published:** 2019-07-26

**Authors:** Tatsuaki Goto, Hisao Homma, Atsushi Kaida, Masahiko Miura

**Affiliations:** Department of Oral Radiation Oncology, Graduate School of Medical and Dental Sciences, Tokyo Medical and Dental University, 1-5-45 Yushima, Bunkyo-ku, Tokyo, Japan

**Keywords:** hypoxia, reoxygenation, DNA double-strand breaks, homologous recombination, WEE1

## Abstract

Hypoxia/reoxygenation (H/R) treatment reportedly induces DNA damage response (DDR), including DNA double-strand break (DSB) repair and G2 arrest, resulting in reduction of clonogenic survival. Because WEE1 plays a key role in the G2/M checkpoint along with CHK1/2, we investigated the effect of WEE1 inhibition on H/R-induced DDR using HeLa cells. The H/R treatment combined with WEE1 inhibitor abrogated G2 arrest, subsequently leading to the cells entering the M phase, and finally resulting in mitotic catastrophe after prolonged mitosis. Colony-forming assay showed an enhanced decrease in the surviving fraction and the focus formation of BRCA1 was significantly reduced. We demonstrate for the first time that WEE1 inhibition enhances H/R-induced cell death accompanied by mitotic catastrophe and that the process may be mediated by homologous recombination.

## INTRODUCTION

The phenomenon of hypoxia/reoxygenation (H/R) is often observed in solid tumors during the naturally occurring process of periodic occlusion and opening of tumor vessels [1]. Hammond *et al.* reported that DNA double-strand breaks (DSBs) are generated and cell cycle checkpoints are activated following H/R treatment [2]. These results raise a possibility that acutely hypoxic cells could be targeted by taking advantage of DNA damage response (DDR) occurring after the reoxygenation. It is thus important to characterize the properties of such DDR.

We have previously reported that H/R induces DSBs and that two cell fractions showing different cell cycle kinetics are detected 24 h after H/R treatment in HeLa cells, namely those cells remaining in G2 phase and harboring plenty of DSBs and those released from G2 arrest and harboring fewer DSBs [3]. In that study, we employed the fluorescent ubiquitination-based cell cycle indicator (Fucci), a cell cycle-visualizing system [4]. It allowed us to isolate the two different cell fractions by flow cytometric sorting, which revealed that the latter fraction exhibits significantly higher survival than the former [3]. If the observed G2 arrest is linked to DSB repair like the process occurring after irradiation [5], G2 arrest-releasing agents should enhance cell killing. Indeed, CHK1 inhibition reportedly enhances sensitivity to H/R [2].

Because WEE1 plays a key role in the G2/M checkpoint along with CHK1/2, herein we aimed to investigate the effect of WEE1 inhibition on H/R-induced DDR in HeLa cells using the WEE1 inhibitor, MK-1775, which relieves G2 arrest [6] via inhibition of the Y15 phosphorylation of CDK1 [5], as well as to assess the involvement of homologous recombination (HR) in the process.

## MATERIALS AND METHODS

### Cell line and culture conditions

HeLa cells expressing the Fucci probes (HeLa-Fucci cells) were provided by RIKEN BRC through the National Bio-Resource Project of MEXT, Japan. HeLa cells were obtained from the Health Bio-Resources Bank (Sendai, Japan). Cells were maintained at 37°C, in a 5% CO_2_ humidified atmosphere, in Dulbecco’s modified Eagle’s medium (Sigma-Aldrich, St. Louis, MO, USA) supplemented with 10% fetal bovine serum, 100 IU/mL penicillin, and 100 μg/mL streptomycin.

### Hypoxia/reoxygenation (H/R) treatment

We established hypoxic conditions using a combination of plastic culture dishes and an AnaeroPack-Anaero 5% system (Mitsubishi Gas Chemical, Tokyo, Japan) and the level of hypoxia was set to pO_2_ < 0.1% as described previously [3]. The reoxygenation was initiated by opening the pack and transferring the culture dishes into the above-described normoxic growth conditions (pO_2_ = 21%).

### Drug treatment

Cells were treated with the WEE1 inhibitor MK-1775 (Axon Medchem, Groningen, The Netherlands) at the concentration of 120 or 300 nM immediately after reoxygenation following hypoxic treatment for 24 h. Fucci fluorescence and immunofluorescence of cells were observed at the indicated times after reoxygenation by flow cytometry and time-lapse imaging as described below. For colony-forming assay, the inhibitor treatment time was 24 h.

### Time-lapse imaging

Treated cells were held in an incubation chamber at 37°C in a humidified atmosphere containing 5% CO_2_ (Tokai Hit, Fujinomiya, Japan) as described previously [3, 7]. Fluorescence images were acquired using a BIOREVO BZ-9000 fluorescence microscope (KEYENCE, Osaka, Japan).

### Fluorescence immunostaining

At the indicated times after reoxygenation, the cells were fixed in 4% paraformaldehyde in PBS for 15 min. After extensive washing in PBS, the cells were subjected to immunostaining and analyzed as described previously [3], except that primary monoclonal antibody against BRCA1 (1:200; SC-6954; Santa Cruz Biotechnology, Dallas, TX, USA) was used.

### Flow-cytometric analysis

The treated cells were trypsinized and centrifuged, and the pellets were washed in PBS. The cells were fixed in 4% paraformaldehyde in PBS for 15 min on ice, and then incubated with a monoclonal antibody against phospho-histone H3 (S10) (1:50; Cell Signaling Technologies, Beverly, MA, USA) for 1 h on ice and then with Alexa Fluor 647-conjugated anti-rabbit IgG (1:500; Invitrogen, Carlsbad, CA, USA) for 30 min. For nuclear counter-staining, the cells were stained with Hoechst33342(1:1000) (Invitrogen) for 30 min. After staining, all samples were washed in PBS. Finally, single-cell suspensions were passed through a nylon mesh. Each sample was analyzed on a FACSCanto II cytometer (Becton Dickinson, Franklin Lakes, NJ) using the FlowJo software (Tree Star, Ashland, OR).

### Colony-forming assay

Cells were treated with MK1775 (120 and 300 nM) for 24 h or incubated without treatment under normoxic conditions after finishing hypoxic treatment for 24 h. Immediately after the treatment, the treated cells were seeded on 60 mm plastic dishes (100 cells per dish). After 7–14 days, the cells were washed twice with PBS, fixed with 4% paraformaldehyde, and stained with 0.05% crystal violet. Colonies consisting of more than 50 cells were counted. All experiments were performed in triplicate and repeated three times.

### Statistical analysis

Mean values were compared statistically using the two-tailed *t*-test or one way ANOVA with *post hoc* Tukey’s multiple comparison test. *P*-values of <0.05 were considered statistically significant.

## RESULTS AND DISCUSSION

### WEE1 inhibition abrogates H/R-induced G2 arrest, resulting in mitotic catastrophe

We have previously reported that H/R treatment induces apparent G2 arrest in HeLa-Fucci cells [3]. Our previous results showed that most cells are arrested in G1 phase in terms of DNA content immediately after hypoxic treatment. Then, the cell cycle progressed through the S phase and reached the G2 phase, wherein arrest was clearly observed up to around 14 h after the treatment [3]. These results prompted us to examine the effect of G2 arrest-releasing agents on the cell cycle kinetics. WEE1 phosphorylates Y15 of CDK1 and negatively regulates transition from the G2 to the M phase in the cell cycle progression [5]. The WEE1 inhibitor MK1775 is known to release the DNA damage-induced G2 arrest [6]. Thus, we employed the inhibitor and the effect for G2 arrest kinetics was analyzed in combination with the M phase marker phosphorylated histone H3 (pHH3).

**Fig. 1.**
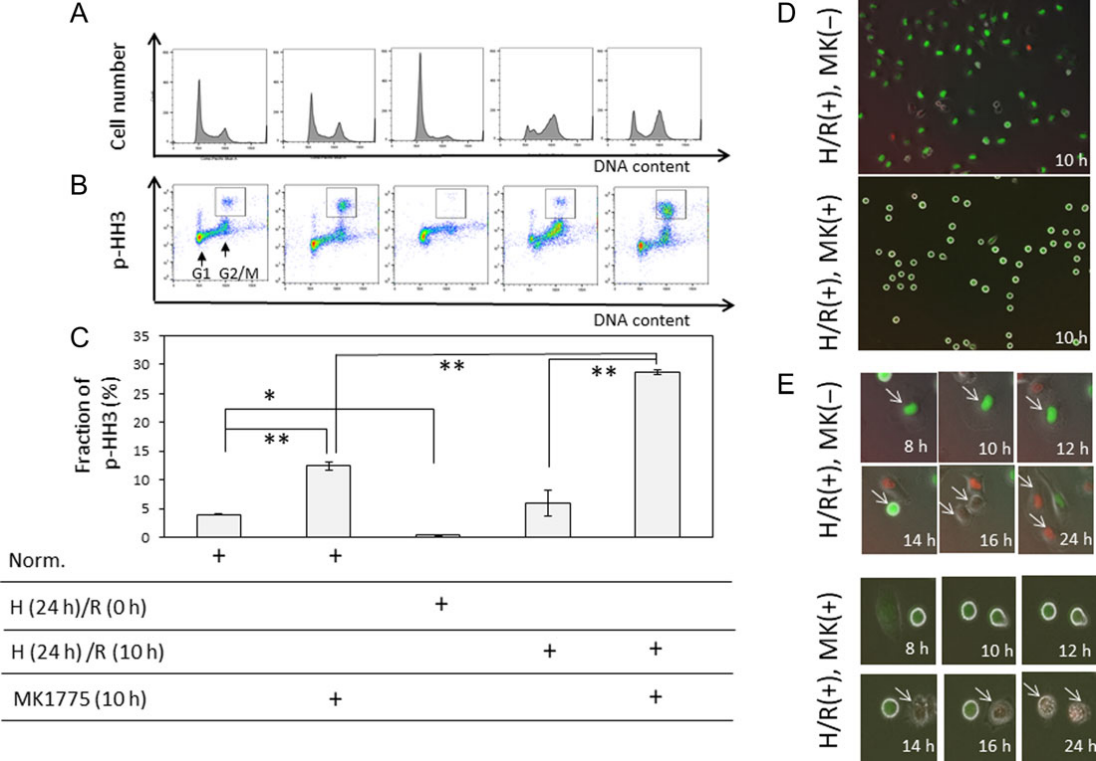
Cell cycle kinetics and time-lapse imaging of Fucci fluorescence after hypoxia/reoxygenation (H/R) alone or H/R followed by WEE1 inhibitor treatment. Flow cytometric analysis of DNA content (Hoechst) (**A**) and phosphorylated histone H3 (p-HH3) (**B**). The square represents the p-HH3-positive fraction. (**C**) Quantitative analysis of pHH3-positive fractions in Fig. 1**B**. Cells were treated as indicated. Cells were subjected to hypoxic treatment for 24 h and reoxygenated for 10 h in the absence or presence of 300 nM MK1775. In some experiments, cells were treated under normoxic conditions. The cells were then prepared for flow cytometric analysis. Data represent mean values ± SD from three independent experiments. *P < 0.05, **P < 0.01. Norm. = normoxia. Time-lapse imaging of Fucci fluorescence (**D**, lower magnification; **E**, higher magnification). Time-lapse images were acquired at the indicated times after reoxygenation in the absence (upper panels) or presence (lower panels) of 300 nM MK1775. The arrows in D (upper panels) indicate G2 arrested cells (green) that later enter the M phase (14 h) and then divide normally into two cells (red cells, 16 and 24 h). Arrows in E indicate cells that died in the M-phase.

Figure 1A and B present the results of DNA content analysis and 2D-analysis of DNA content/pHH3, respectively, following various treatments as indicated. MK1775 (300 nM) increased the M-phase fraction even under normoxic conditions. Pedigree analysis showed that the M-phase duration elongated to about 2 h (normal M-phase duration: ~1 h), but cells normally divided (data not shown). Immediately after exposure to hypoxia for 24 h, fractions of S/G2/M phases decreased and the G2/M fraction similarly increased 10 h after reoxygenation in the absence or presence of MK1775. However, in the presence of MK1775, the M-phase fraction increased 10 h after reoxygenation, indicating the release from G2 arrest, and in this fraction, the pHH3-positive fraction was significantly higher than that observed in the presence of the inhibitor under normoxic conditions (Fig. 1C, *P* < 0.01).

Furthermore, time-lapse imaging of Fucci fluorescence (Fig. 1D) allowed visualization of red and green fluorescence emitted in the G1 and S/G2/M phases, respectively [4]. Most of the cells showed green fluorescence 10 h after H/R treatment, representing G2 arrest, which was consistent with the above described findings (Fig. 1D, upper panel). Thereafter, some red cells, indicating release from G2 arrest, were observed, and no cell death was detected around 24 h after the H/R treatment (Fig. 1E, upper panel). On the contrary, many round green cells characteristic of the M phase were observed 10 h after the combined treatment (Fig. 1D, lower panel). Thereafter, the M phase was abnormally maintained for >5 h (the normal duration of the M phase in HeLa-Fucci cells being ~1 h, [8]) and followed by cell death during mitosis 14–24 h after the H/R treatment (Fig. 1E, lower panel), a phenomenon termed mitotic catastrophe [9]. Similar effects were obtained in another HeLa cell line, HeLa-H2B-GFP, independently established in Dr Saya’s laboratory (Keio Univ.) [9], which showed a lower growth rate (18 h for HeLa-Fucci cells vs 24 h for HeLa-H2B-GFP cells). A human oral cancer cell line, SAS, with a p53 gene mutation [10] and diploid fibroblasts expressing telomerase reverse transcriptase (TERT), BJ1-hTERT [8], did not exhibit a significant M-phase arrest like the HeLa cell lines treated with H/R plus MK1775 (See Supplementary Fig. 1).

**Fig. 2.**
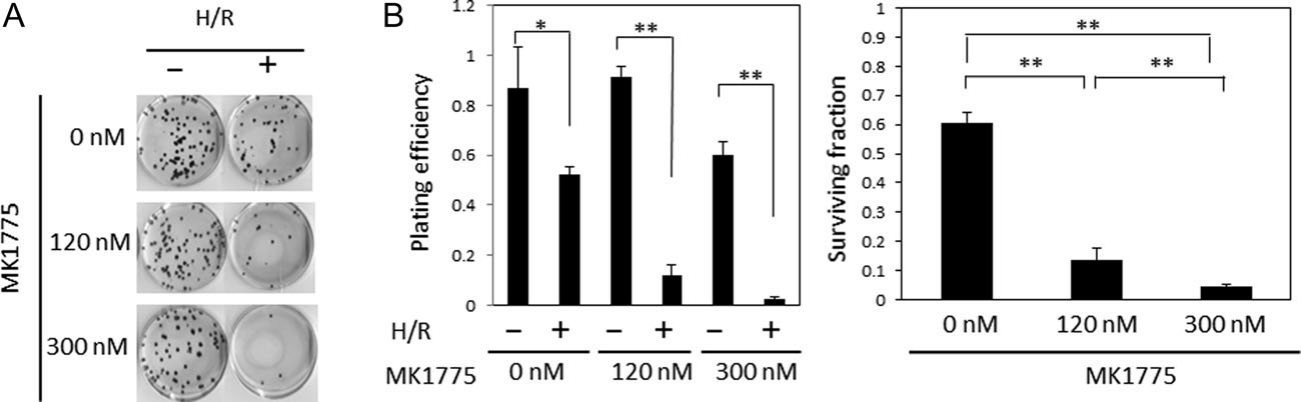
Clonogenic survival after treatment with H/R alone and H/R plus MK1775. **(A)** Photographs of colonies formed in the plates after treatment with H/R alone or H/R plus MK1775. Cells were treated with MK1775 (120 and 300 nM) for 24 h in normoxic conditions after finishing hypoxic treatment for 24 h and subjected to colony-forming assay immediately after the treatment. **(B)** Plating efficiencies (left panel) and surviving fractions (right panel) of cells after treatment with H/R alone or H/R plus MK1775. Surviving fractions were calculated by normalizing plating efficiencies from the data in the left panel. Data represent mean values ± SD from three independent experiments. *P < 0.05, **P < 0.01.

We also examined the effect of the WEE1 inhibitor on H/R-induced clonogenic survival in HeLa-Fucci cells. After treatment with 120 and 300 nM MK1775, the cells were subjected to colony-forming assay, and the results showed that clonogenic survival was significantly reduced in a dose-dependent manner (Fig. 2). Significant difference was not detected between cells treated with H/R alone and H/R plus 300 nM MK1775 in SAS cells (0.8 ± 0.3, *n* = 3; 0.4 ± 0.3, *n* = 3) or BJ1-hTERT cells (0.7 ± 0.1, *n* = 3; 0.4 ± 0.3, *n* = 3) in terms of survival fraction. Thus, further analysis is required to confirm whether the observed findings are attributable to p53 functions or a HeLa cells-specific phenomenon.

### WEE1 inhibition inhibits focus formation of BRCA1 in H/R-treated cells

**Fig. 3.**
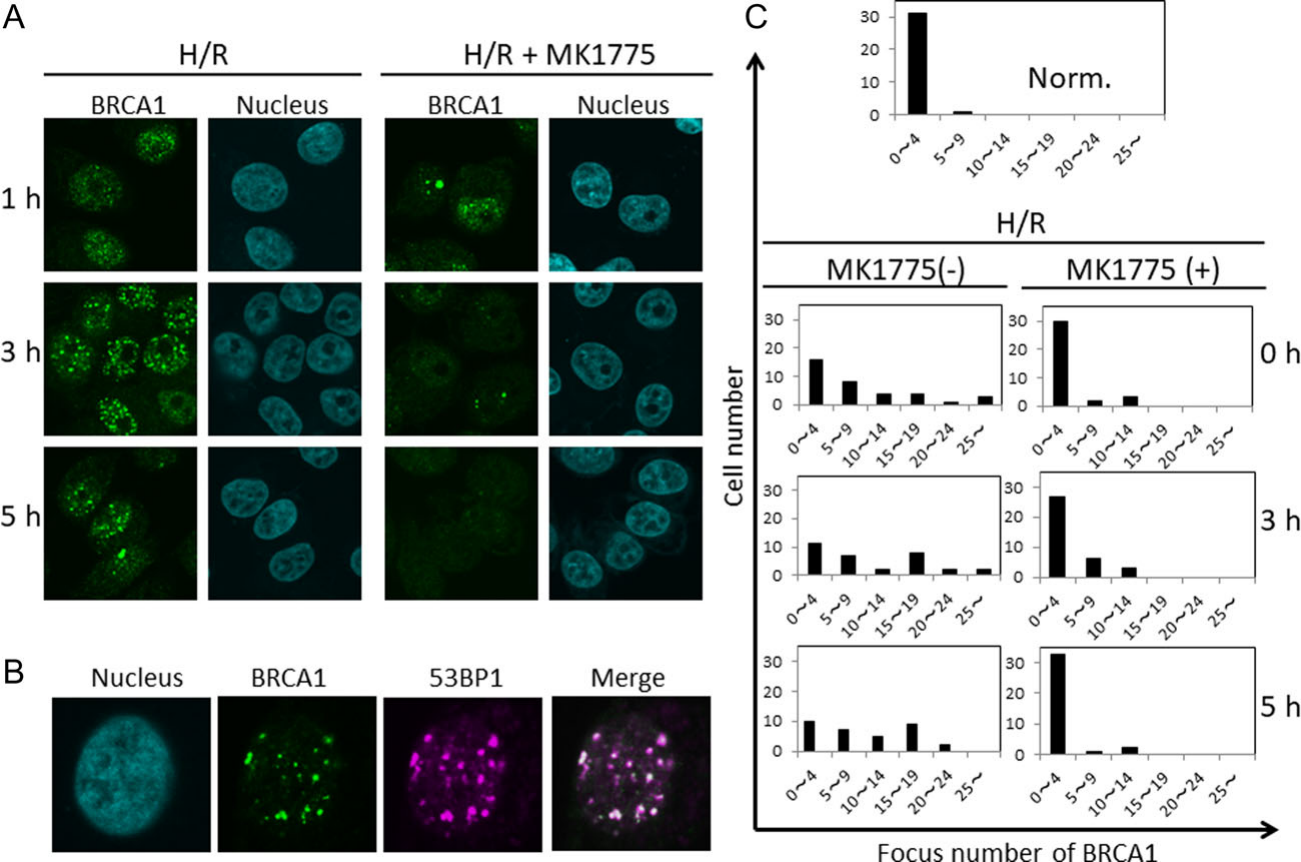
Fluorescence immunostaining for focus formation of BRCA1 in cells after treatment with H/R alone or H/R plus MK1775. (**A**) Fluorescence immunostaining of BRCA1 and counter-staining of nuclei with Hoechst. The cells were fixed at the indicated times after reoxygenation and prepared for immunostaining. (**B**) Double fluorescence immunostaining of BRCA1 and 53BP1, and counter-staining of nuclei with Hoechst. The cells were fixed 7 h after reoxygenation and prepared for immunostaining. Norm. = normoxia. (**C**) Histograms for BRCA1 focus number. Cells with different focus numbers were classified into six groups: 0–4, 5–9, 10–14, 15–19, 20–24, and ≥25 and shown as histograms. At least 30 cells were counted for each time point. A representative result is presented from three independent results.

WEE1 inhibition reportedly results in suppression of HR following irradiation [11]. Therefore, we attempted to examine the effect of MK1775 on BRCA1 relocation to DSB sites, a key process in HR [12]. Figure 3A shows immunofluorescence staining for BRCA1 in cells treated with H/R alone or with H/R and MK1775 combined. Up to 5 h after the treatment, clear focus formation of BRCA1 was observed in cells treated with H/R alone but not in cells treated with the combination. BRCA1 foci were colocalized with 53BP1 foci that are also recruited to DSB sites (Fig. 3B) [13]. Histograms for focus number of BRCA1 quantitatively confirmed the results (Fig. 3C). These findings suggest that HR may be involved in repair of DSBs induced by the H/R treatment and that inhibition of HR, through WEE1 inhibition, may result in mitotic catastrophe. Krajewska *et al.* reported that WEE1 inhibition results in the activation of CDK1, which in turn phosphorylates S3291 of BRCA2, thus contributing to HR suppression [14]. Given that BRCA1 functions at the upstream regulator of BRCA2 [11], it is intriguing to speculate the existence of other mechanisms that link WEE1-associated events with BRCA1. Chronic hypoxia has also been reported to inhibit the expression of HR proteins, including BRCA1/2, which compromises the radioresistance of chronically hypoxic cells [15]. The possibility that WEE1 inhibition somehow enhances hypoxia-induced suppression of BRCA1 protein expression could not be ruled out. These questions thus warrant further investigation.

## CONCLUSION

In this study, we demonstrate for the first time that WEE1 inhibition suppresses BRCA1 relocation to DSB sites, followed by mitotic catastrophe in H/R-treated HeLa cells. The present study may shed additional light on understanding the DDR occurring after H/R treatment.

## Supplementary Material

rrz045_SuppleR1

## References

[1] Hall EJ, Giaccia AJ Oxygen effect and reoxygenation In: Hall EJ, Giaccia AJ (ed). Radiobiology for the radiologist. 7th edn Philadelphia: Lippincott Williams & Wilkins, 2012, 86–103.

[2] Hammond EM, Freiberg RA, Giaccia AJ The roles of Chk1 and Chk2 in hypoxia and reoxygenation. Cancer Lett 2006;238:161–7. 16085356 10.1016/j.canlet.2005.06.029

[3] Goto T, Kaida A, Miura M Visualizing cell cycle kinetics after hypoxia/reoxygenation in HeLa cells expressing fluorescent ubiquitination-based cell cycle indicator (Fucci). Exp Cell Res 2015;339:389–96. 26500111 10.1016/j.yexcr.2015.10.019

[4] Sakaue-Sawano A, Kurosawa H, Morimura T et al Visualizing spatiotemporal dynamics of multicellular cell-cycle progression. Cell 2008;132:487–98. 18267078 10.1016/j.cell.2007.12.033

[5] de Gooijer MC, van den Top A, Bockaj I et al The G2 checkpoint-a node-based molecular switch. FEBS Open Bio 2017;7:439–55. 10.1002/2211-5463.12206 PMC5377395 28396830

[6] Hirai H, Iwasawa Y, Okada M et al Small-molecule inhibition of Wee1 kinase by MK-1775 selectively sensitizes p53-deficient tumor cells to DNA-damaging agents. Mol Cancer Ther 2009;8:2992–3000. 19887545 10.1158/1535-7163.MCT-09-0463

[7] Kaida A, Miura M Visualizing the effect of hypoxia on fluorescence kinetics in living HeLa cells using the fluorescent ubiquitination-based cell cycle indicator (Fucci). Exp Cell Res 2012;318:288–97. 22079518 10.1016/j.yexcr.2011.10.016

[8] Tsuchida E, Kaida A, Pratama E et al Effect of X-irradiation at different stages in the cell cycle on individual cell-based kinetics in an asynchronous cell population. PLoS One 2015;10:e0128090. 26086724 10.1371/journal.pone.0128090 PMC4472673

[9] Nitta M, Kobayashi O, Honda S et al Spindle checkpoint function is required for mitotic catastrophe induced by DNA-damaging agents. Oncogene 2004;23:6548–58. 15221012 10.1038/sj.onc.1207873

[10] Jia LQ, Osada M, Ishioka C et al Screening the p53 status of human cell lines using a yeast functional assay. Mol Carcinog 1997;19:243–53. 9290701 10.1002/(sici)1098-2744(199708)19:4<243::aid-mc5>3.0.co;2-d

[11] Kamak D, Engelke CG, Parsels LA et al Combined inhibition of Wee1 and PARP1/2 for radiosensitization in pancreatic cancer. Clin Cancer Res 2014;20:5085–96. 25117293 10.1158/1078-0432.CCR-14-1038 PMC4184968

[12] Bekker-Jensen S, Mailand N Assembly and function of DNA double-strand break repair foci in mammalian cells. DNA Repair (Amst) 2010;9:1219–28. 21035408 10.1016/j.dnarep.2010.09.010

[13] Davis AJ, Chen PC, Chen DJ DNA-PK:A dynamic enzyme in a versatile DSB repair pathway. DNA Repair (Amst) 2014;17:21–9. 24680878 10.1016/j.dnarep.2014.02.020 PMC4032623

[14] Krajewska M, Heijink AM, Bisselink YJ et al Forced activation of Cdk1 via wee1 inhibition impairs homologous recombination. Oncogene 2013;32:3001–8. 22797065 10.1038/onc.2012.296

[15] Chan N, Koritzinsky M, Zhao H et al Chronic hypoxia decreases synthesis of homologous recombination proteins to offset chemoresistance and radioresistance. Cancer Res 2008;68:605–14. 18199558 10.1158/0008-5472.CAN-07-5472

