# Supplementary figures and images for: WEE1 inhibition enhances sensitivity to hypoxia/reoxygenation in HeLa cells
Source: J Radiat Res. 2019 Jul 26;60(5):709–13. doi: 10.1093/jrr/rrz045 (PMC6805980; doi:10.1093/jrr/rrz045)

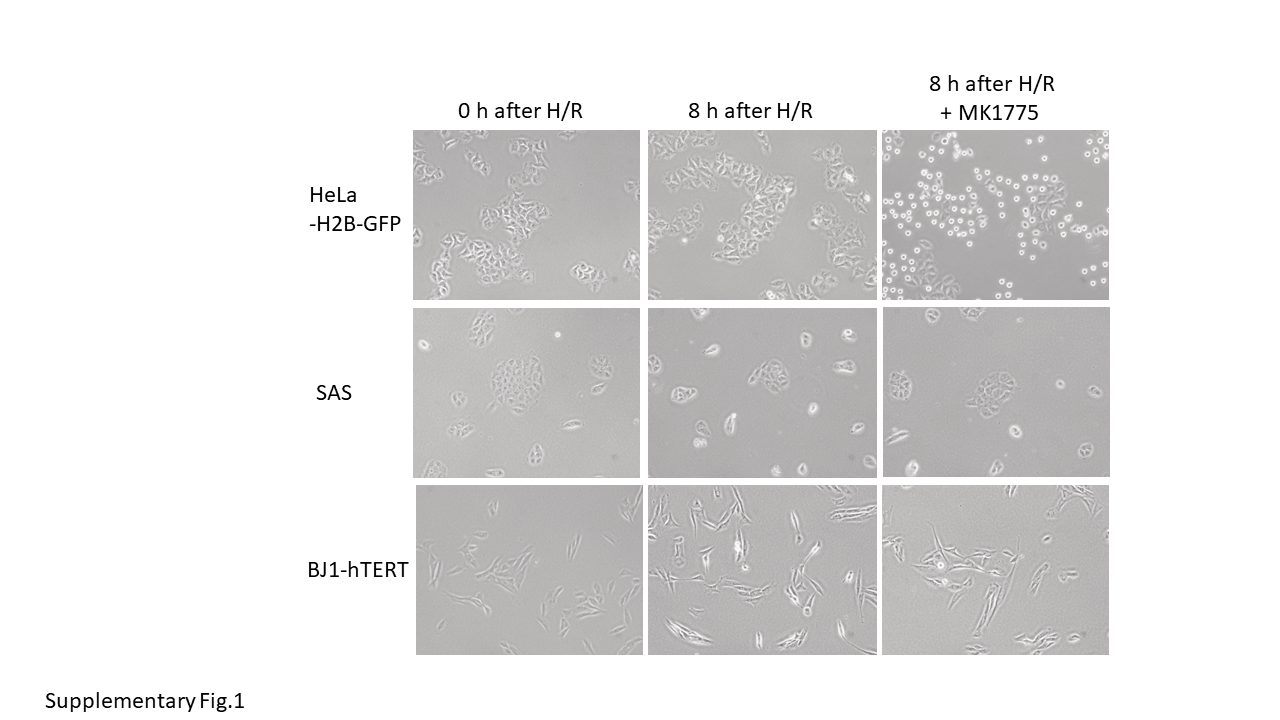

Supplement: rrz045_SuppleR1 [file rrz045_suppler1.png]
